# Comparison of neoadjuvant nab‐paclitaxel plus immunotherapy versus paclitaxel plus immunotherapy for esophageal squamous cell carcinoma

**DOI:** 10.1111/1759-7714.14795

**Published:** 2023-02-14

**Authors:** Yafan Yang, Haomiao Li, Xiankai Chen, Jianjun Qin, Yong Li, Yaxing Shen, Ruixiang Zhang, Xiaozheng Kang, Zhen Wang, Qingfeng Zheng, Peng Luo, Yin Li, Jie He

**Affiliations:** ^1^ Department of Thoracic Surgery National Cancer Center/National Clinical Research Center for Cancer/Cancer Hospital, Chinese Academy of Medical Sciences and Peking Union Medical College Beijing China; ^2^ Department of Thoracic Surgery Henan Cancer Hospital Zhengzhou City China; ^3^ Department of Thoracic Surgery Zhongshan Hospital, Fudan University Shanghai China

**Keywords:** immunotherapy, nab‐, paclitaxel, Paclitaxel esophageal squamous cell carcinoma

## Abstract

**Background:**

This study aimed to compare the feasibility of nab‐paclitaxel plus platinum‐based chemotherapy (nabTP) versus paclitaxel plus platinum‐based chemotherapy (TP) with immune checkpoint inhibitors (ICIs) as a neoadjuvant modality for locally resectable esophageal squamous cell carcinoma (ESCC).

**Methods:**

Between April 2019 and March 2022, we identified ESCC patients who received neoadjuvant immunotherapy with both nabTP (*n* = 213) and TP (*n* = 98) at our institution and Henan Cancer Hospital. The patients in the ICIs‐nabTP and ICIs‐TP groups were pair‐matched (1:1) for tumor location, sex, smoking, drinking, clinical T and N stage. The primary endpoint was the hazard of 30‐day major postoperative complications. Second, logistic models were applied to estimate the risk factors for pathological complete response (pCR) rate.

**Results:**

All patients underwent esophagectomy with R0 resection. A statistically significant increase in the risk of developing major pulmonary (odds ratio [OR], 1.182; 95% confidence interval [CI]: 0.530–2.635; *p* = 0.683), anastomotic (OR, 1.881; 95% CI: 0.607–5.830; *p* = 0.267), cardiac (OR, 1.000; 95% CI: 0.426–2.349; *p* = 1.000) complications after neoadjuvant immunotherapy plus nabTP was not observed. The median interval to surgery was 39 days in the ICIs‐nabTP group versus 44 days in the ICIs‐TP group (*p* = 0.119). There was no 30‐day mortality in each group. However, there was a slight difference in the 30‐day readmission rate (*p* = 0.043) and the incidence of hydropneumothorax (*p* = 0.027) between the two groups. The pCR rates of the ICIs‐nabTP and ICIs‐TP group were 36.7 and 21.4%, respectively (*p* = 0.018).

**Conclusions:**

It appears to be feasible to add immunotherapy to nabTP regimen for locally advanced ESCC. Compared with TP, nabTP plus ICIs can achieve a better pCR rate in ESCC.

## Graphical abstract

The analysis was first conducted to compare the safety and feasibility of nab‐paclitaxel plus platinum‐based chemotherapy (nabTP) versus paclitaxel plus platinum‐based chemotherapy (TP) with immune checkpoint inhibitors (ICIs) as a neoadjuvant modality for locally resectable esophageal squamous cell carcinoma (ESCC).

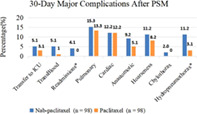

## INTRODUCTION

Esophageal cancer (EC) ranks the seventh in most commonly diagnosed cancer (604 000 new cases) and the sixth in mortality (544 000 deaths) worldwide in 2020.^1^ Although neoadjuvant chemoradiotherapy (nCRT) followed by surgery has been the standard treatment option for resectable EC patients,^2^, ^3^ the effectiveness of nCRT in patients with EC is controversial.^4^, ^5^, ^6^, ^7^ The Chinese NEOCRTEC5010 trial proves that nCRT plus surgery improves survival among ESCC patients.^2^ However, some studies fail to confirm the superiority of nCRT over neoadjuvant chemotherapy (nCT) for resectable carcinoma of the esophagus.^8^, ^9^ It is therefore important to establish novel treatment strategies for ESCC to further improve short‐ and long‐term effects.

Recently, ICIs have gained popularity in the neoadjuvant setting, but few studies have examined surgical safety following their use.^10^, ^11^ Several feasibility trials show that combining nCRT/nCT with ICIs for patients with resectable EC is safe and feasible without compromising surgical outcomes.^12^, ^13^, ^14^, ^15^, ^16^, ^17^, ^18^ However, we found some variability in tumor remission rates for the treatment of nCT with ICIs, from 18.8% in the ICIs‐TP group^17^ to less than 50% in the ICIs‐nabTP group.^15^ Thus, nab‐paclitaxel, a specific chemotherapeutic agent, may have a better treatment outcome in combination with immunotherapy.

Nab‐paclitaxel is a new generation formulation of paclitaxel, which is bound to albumin and a solvent‐free form of paclitaxel.^19^, ^20^ It has several advantages over paclitaxel, including its ability to deliver higher doses within shorter infusion times, and its elimination of premedication. Nab‐paclitaxel also enhances the transport of paclitaxel across endothelial cells and delivers more paclitaxel to tumors.^20^ Nab‐paclitaxel is also less toxic and more potently antitumor than solvent‐based paclitaxel, according to several previousl clinical studies on breast cancer, non‐small cell lung carcinoma, pancreatic cancer, melanoma, and ovarian cancer.^21^, ^22^, ^23^, ^24^, ^25^ Particularly for advanced ESCC cases, nabTP as a first‐line therapy may improve survival with an increased response rate.^26^ In addition, nab‐paclitaxel based neoadjuvant chemotherapy has been shown to produce positive results for locally advanced ESCC.^27^ Nevertheless, the comparison of nabTP‐ICIs and TP‐ICIs schemes is still lacking. In this study, we summarize the comparative results of neoadjuvant ICIs with nabTP versus TP for locally advanced ESCC patients using perioperative outcomes and 30‐day complications.

## METHODS

### Patients

We consulted our prospective surgical database to identify patients with thoracic ESCC who underwent neoadjuvant chemoimmunotherapy and surgery between April 2019 and March 2022 at the National Cancer Hospital and Henan Tumor Hospital, China.

The following inclusion criteria were used: (1) Pathologically confirmed primary thoracic ESCC; (2) clinical stage T1N1–3 or T2‐4aN0‐3 (AJCC TNM classification, eighth edition); (3) patients who underwent radical R0 resection; (3) patients who received neoadjuvant chemotherapy and immunotherapy (TP/nabTP plus ICIs) and (4) patients who completed clinicopathological date for analysis. The study excluded patients who had cervical EC or a palliative procedure, as well as those with missing data regarding neoadjuvant regimens or postoperative complications. Where possible, the nabTP‐ICIs subjects were matched to the TP‐ICIs group to remove heterogeneity between the two groups. The two cohorts were pair‐matched (1:1) for tumor location, sex, smoking, drinking, clinical T and N stage. All patients underwent radical resection of EC, and the follow‐up time reached 30 days after operation.

### Neoadjuvant treatment

All patients received at least two cycles of paclitaxel/nab‐paclitaxel plus platinum‐based chemotherapy. The TP/nabTP regimen comprised platinum‐based drugs (cisplatin or nedaplatin: 75 mg/m^2^ IV Q3W; carboplatin: area under the curve [AUC] 5 Q3W) and nab‐paclitaxel (250 mg/m2 IV Q3W) or paclitaxel (175 mg/m^2^ IV Q3W). ICI agents were administered intravenously at a dose of 200 mg every 3 weeks, and mainly included camrelizumab, toripalimab, pembrolizumab or sintilimab. Some of the cases receiving ICIs were included in our randomized clinical trial (ChiCTR2000040034). The usage and dosage of chemotherapeutics and ICIs were determined according to the case's condition.

### Surgical procedure and outcome measurements

The EC was treated by minimally invasive McKeown esophagectomy with extensive two‐field lymphadenectomy in all patients. All cases were reconstructed using a gastric conduit. Two independent local pathologists assessed the objective pathological response and the pCR was defined as the absence of tumor cells in the specimen. The primary endpoint was the hazard of 30‐day postoperative complications (Clavien‐Dindo classification system grade ≥ 3). All complications were classified into four main categories. Pneumonia and respiratory failure requiring reintubation were defined as pulmonary complications. Cardiac complications mainly included myocardial infarction, heart failure and arrhythmia. The most common anastomotic complications were leak, fistula, and dehiscence. Remaining complications included pleural effusion or aerothorax requiring drainage of thoracic puncture, chyle leak and hoarseness. Secondary endpoints were the number of retrieved lymph nodes, interval to surgery, 30‐day readmission, unplanned transfer to ICU rate, blood transfusion rate and pCR rate. From the end of neoadjuvant therapy to the date of surgery, the interval to surgery was measured.

### Statistical analysis

Characteristics are summarized as frequency compared among two groups using either a chi‐squared or Fisher's exact test (categorical variables). An independent sample *t*‐test was applied to estimate the parameters, including interval to surgery, operative time, postoperative hospitalization days, the number of retrieved lymph nodes. In order to calculate risk factors for pCR, logistic regression analysis was used. Statistical significance was determined by two‐sided *p* < 0.05. SPSS version 23.0 was used for all statistical analyses.

## RESULTS

### Patients

**FIGURE 1:**
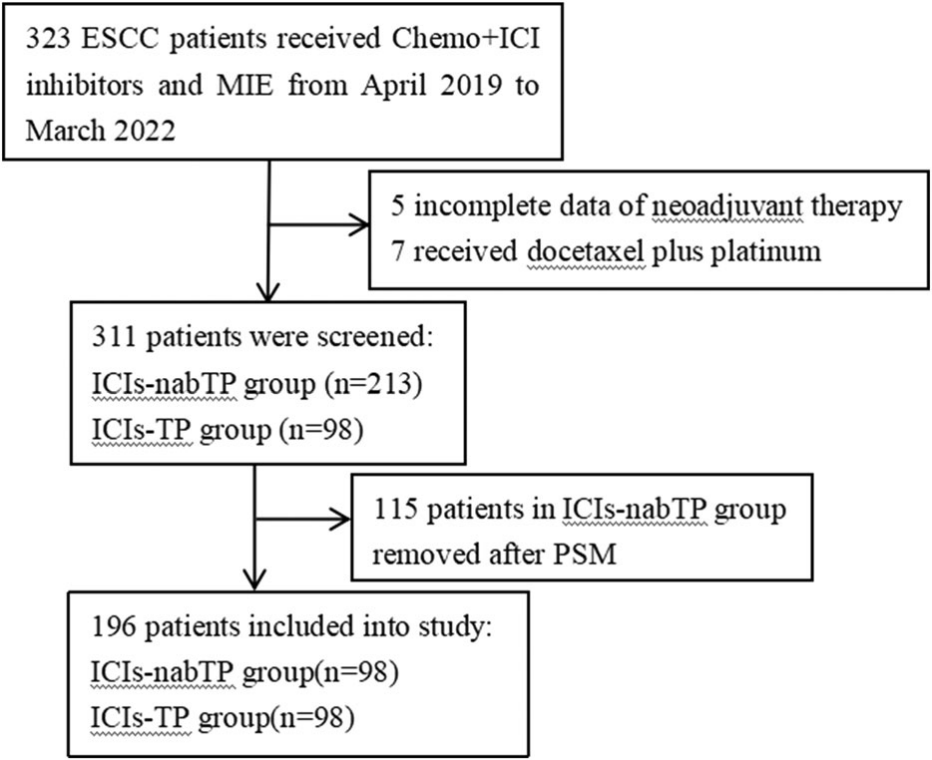
Flowchart of patient selection.

From April 2019 to March 2022, we identified a total of 323 in patients who had completed McKeown resection following neoadjuvant therapy in our hospital and Henan Cancer Hospital. A total of 311 patients with locally advanced ESCC received neoadjuvant anti‐PD‐1/PD‐L1 immunotherapy and nabTP/TP chemotherapy. Of 311 patients, 98 patients were included in the TP‐ICIs group, and 213 patients in the nabTP‐ICIs group (Figure 1). Finally, the 98 patients in the ICIs plus nabTP cohort were pair‐matched (1:1) with cases in the TP‐ICIs group for tumor location, sex, clinical stage, smoking and drinking history. Patient characteristics are summarized in Table 1 before and after propensity score‐matched (PSM) analysis. Both groups had a majority of male patients. Most tumors were located in the middle and lower thoracic esophagus, with moderate to poor differentiation. The distribution of major comorbidities were well balanced between both groups.

**TABLE 1:** Baseline characteristics of patients

Characteristic	Entire study cohort			Matched study cohort		
	Nab‐paclitaxel (%) (*n* = 213)	Paclitaxel (%) (*n* = 98)	*p*	Nab‐paclitaxel (%) (*n* = 98)	Paclitaxel (%) (*n* = 98)	*p*
Age (<62 years)	100 (46.9)	49 (50.0)	0.617	42 (42.9)	49 (50.0)	0.316
BMI (<23.3)	94 (44.1)	53 (54.1)	0.103	45 (45.9)	53 (54.1)	0.253
Man	178 (83.6)	87 (88.8)	0.229	79 (80.6)	87 (88.8)	0.112
Smoking	139 (65.3)	61 (62.2)	0.606	59 (60.2)	61 (62.2)	0.769
Drinking	142 (66.7)	49 (50.0)	0.005*	52 (53.1)	49 (50.0)	0.668
T stage			0.248			0.621
cT1‐2	32 (15.0)	10 (10.2)		8 (8.2)	10 (10.2)	
cT3‐4	181 (85.0)	88 (89.8)		90 (91.8)	88 (89.8)	
N stage			0.040*			0.830
cN0‐1	166 (77.9)	86 (87.8)		85 (86.7)	86 (87.8)	
cN2‐3	47 (22.1)	12 (12.2)		13 (13.3)	12 (12.2)	
Location			0.130			0.956
Upper	41 (19.2)	18 (18.4)		17 (17.3)	18 (18.4)	
Middle	61 (28.6)	39 (39.8)		38 (38.8)	39 (39.8)	
Lower	111 (52.1)	41 (41.8)		43 (43.9)	41 (41.8)	
Grade			0.116			0.217
G1	12 (5.6)	8 (8.2)		5 (5.1)	8 (8.2)	
G2	95 (44.6)	29 (29.6)		42 (42.9)	29 (29.6)	
G3	83 (39.0)	42 (42.9)		38 (38.8)	42 (42.9)	
Unknown	23 (10.8)	19 (19.4)		13 (13.3)	19 (19.4)	
Chemo interval (≤4 weeks)	43 (20.2)	23 (23.5)	0.511	17 (17.3)	23 (23.5)	0.288
Chemo cycles (2–4)	201 (94.4)	94 (95.9)	0.565	92 (93.9)	94 (95.9)	0.516
ICIs			0.536			0.878
Camrelizumab	75 (35.2)	31 (31.6)		32 (32.7)	31 (31.6)	
Other (toripalimab, pembrolizumab or sintilimab)	138 (64.8)	67 (68.4)		66 (67.3)	67 (68.4)	
Platinum			0.010*			0.138
Cisplatin/nedaplatin	177 (83.1)	92 (93.9)		86 (87.8)	92 (93.9)	
Carboplatin	36 (16.9)	6 (6.1)		12 (12.2)	6 (6.1)	
PPI (yes)	74 (34.7)	23 (23.5)	0.046*	32 (32.7)	23 (23.5)	0.152
Nerve invasion	29 (13.6)	13 (13.3)	0.933	14 (14.3)	13 (13.3)	0.836
Blood vessel invasion	37 (17.4)	24 (24.5)	0.142	20 (20.4)	24 (24.5)	0.493
pCR	63 (29.6)	21 (21.4)	0.133	36 (36.7)	21 (21.4)	0.018*
Pulmonary comorbid	27 (12.7)	12 (12.2)	0.915	12 (12.2)	12 (12.2)	1.000
COPD	0 (0.0)	2 (2.0)	0.036*	0 (0.0)	2 (2.0)	0.155
Emphysema	13 (6.1)	5 (5.1)	0.725	6 (6.1)	5 (5.1)	0.756
Other	14 (6.6)	5 (5.1)	0.615	6 (6.1)	5 (5.1)	0.756
Cardiovascular comorbid	73 (34.3)	32 (32.7)	0.779	34 (34.7)	32 (32.7)	0.762
Hypertension	60 (28.2)	28 (28.6)	0.942	31 (31.6)	28 (28.6)	0.640
Coronary heart disease	11 (5.2)	6 (6.1)	0.730	5 (5.1)	6 (6.1)	0.756
Arrhythmia	15 (7.0)	2 (2.0)	0.071	5 (5.1)	2 (2.0)	0.248
Cerebral stroke	10 (4.7)	6 (6.1)	0.596	4 (4.1)	6 (6.1)	0.516
Hepatic and renal comorbid	10 (4.7)	3 (3.1)	0.716	2 (2.0)	3 (3.1)	0.651
Diabetes	22 (10.3)	3 (3.1)	0.029*	9 (9.2)	3 (3.1)	0.074

* *p* < 0.05.

### Surgical outcomes

**FIGURE 2:**
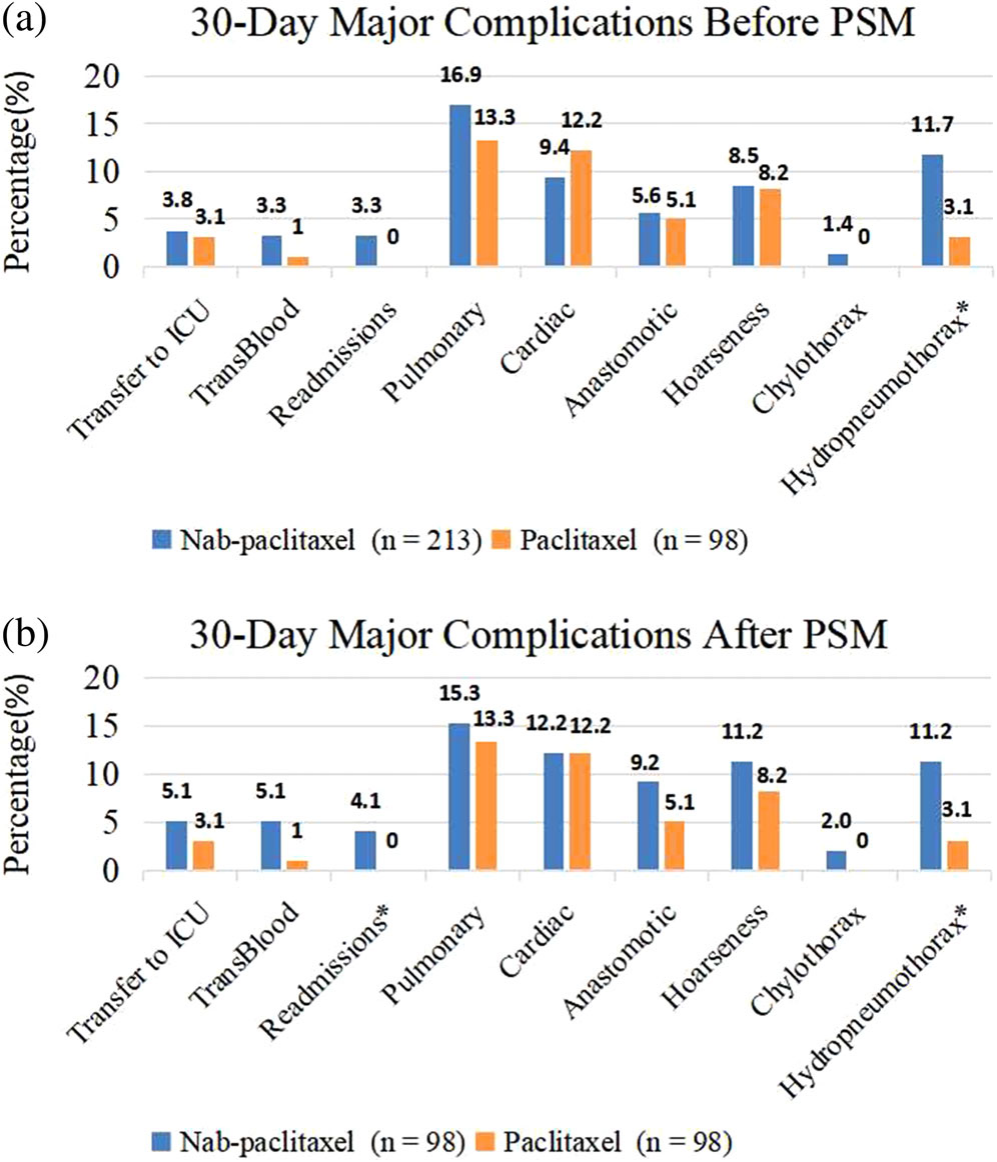
(a) The comparison of ICIs‐nabTP versus ICIs‐TP in 30‐day complications before propensity score‐matching (PSM). (b) The comparison of ICIs‐nabTP versus ICIs‐TP in 30‐day complications after PSM. We compared postoperative short‐term complications between the ICIs‐nabTP group and ICIs‐TP group before and after propensity score‐matched (PSM) analysis. Before PSM analysis, the incidence of hydropneumothorax was slightly higher in the ICIs‐nabTP group than that in the ICIs‐TP group (11.7% vs. 3.1%, *p* = 0.013; Figure 2a). After PSM analysis, the frequencies of both 30‐day readmission and hydropneumothorax were higher in the ICIs‐nabTP group than that in the ICIs‐TP group (4.1% vs. 0.0%, *p* = 0.043; 11.2% vs. 3.1%, *p* = 0.027; Figure 2b). However, irrespective of whether before or after PSM, we found no statistical difference in major complications including heart, lung and anastomosis between the two groups.“*” means *p* < 0.05

The complications within 30‐day before and after PSM are summarized in Figure 2 and Table 2. As part of the ICIs‐TP group, 13 patients (13.3%) had a pulmonary complication, 12 (12.2%) suffered from a cardiac complication, five (5.1%) suffered from an anastomotic complication, and 11 (11.2%) experienced other complications: hoarseness, chylothorax and hydropneumothorax (Table 2). In comparison, among the 98 patients in the ICIs‐nabTP group after 1:1 matching, the incidence of pulmonary, cardiac and anastomotic complications were 15.3, 12.2 and 9.2%, respectively (Table 2; Figure 2). It showed that treatment with neoadjuvant nabTP and ICIs was not related to a statistically increased risk of developing postoperative pulmonary (OR, 1.182; 95% CI: 0.530–2.635; *p* = 0.683), cardiac (OR, 1.000; 95% CI: 0.426–2.349; *p* = 1.000), anastomotic (OR, 1.881; 95% CI: 0.607–5.830; *p* = 0.267) complications. Also, the blood transfusion and unplanned transfer to ICU rates did not differ significantly between two groups (Table 2). There was no 30‐day mortality in each group. However, the frequencies of 30‐day readmission and hydropneumothorax were slightly higher in the ICIs‐nabTP group than that in the ICIs‐TP group (*p <* 0.05, Table 2; Figure 2). Of note, in the ICIs‐nabTP group, 36 (36.7%) patients obtained pCR in the primary tumor and lymph nodes, which was more than the other ICIs‐TP group (*p* = 0.018; Table 1). Therefore, albumin paclitaxel combined with platinum‐based chemotherapy and immunotherapy did not increase the major cardiopulmonary and anastomotic complications in patients with locally advanced ESCC, which appeared to be safe and feasible.

**TABLE 2:** Postoperative outcomes after propensity score‐matching

Variables	Entire study cohort			Matched study cohort		
	Nab‐paclitaxel (%) (*n* = 213)	Paclitaxel (%) (*n* = 98)	*p*	Nab‐paclitaxel (%) (*n* = 98)	Paclitaxel (%) (*n* = 98)	*p*
Unplanned transfer to ICU	8 (3.8)	3 (3.1)	0.758	5 (5.1)	3 (3.1)	0.470
TransBlood	7 (3.3)	1 (1.0)	0.241	5 (5.1)	1 (1.0)	0.097
30‐Day readmissions	7 (3.3)	0 (0.0)	0.069	4 (4.1)	0 (0.0)	0.043*
Pulmonary morbid	36 (16.9)	13 (13.3)	0.414	15 (15.3)	13 (13.3)	0.683
Pneumonia	26 (12.2)	12 (12.2)	0.992	11 (11.2)	12 (12.2)	0.824
Respiratory failure	3 (1.4)	1 (1.0)	0.778	1 (1.0)	1 (1.0)	1.000
Cardiac morbid	20 (9.4)	12 (12.2)	0.441	12 (12.2)	12 (12.2)	1.000
Anastomotic morbid	12 (5.6)	5 (5.1)	0.848	9 (9.2)	5 (5.1)	0.267
Hoarseness	18 (8.5)	8 (8.2)	0.932	11 (11.2)	8 (8.2)	0.469
Chylothorax	3 (1.4)	0 (0.0)	0.238	2 (2.0)	0 (0.0)	0.336
Hydropneumothorax	25 (11.7)	3 (3.1)	0.013*	11 (11.2)	3 (3.1)	0.027*

* *p* < 0.05.

The median interval to surgery was 39 days (range, 20–116 days) in the ICIs‐TP group, and 44 days (range, 11–235 days) in the ICIs‐nabTP group (*p* = 0.119). Median operative time was also comparable: 4.7 h in the ICIs‐TP group versus 4.6 h in the ICIs‐nabTP group (*p* = 0.328). The median number of resected lymph nodes was approximately 36 and median length of hospitalization around 12 days were similar in both groups.

### Univariate and multivariate analysis for pCR

Table 3 shows the results of logistic regression analysis of the pCR. Among baseline parameters, chemotherapy drugs (nab‐paclitaxel vs. paclitaxel), nerve and blood vessel invasions (yes vs. no) were the significant factors influencing pathological remission degree in univariate analysis (Table 3). Meanwhile, we were unable to determine whether the ICI camrelizumab, platinum‐based chemotherapy drug cisplatin or nedaplatin, or whether proton pump inhibitors (PPI) affected, or did not affect, the pathological regression of tumors. Although most of the patients included in the study received 2–4 cycles of chemotherapy and immunotherapy, more than four cycles of neoadjuvant therapy and more than 4 weeks of interval to surgery, were not significantly associated with reduced residual disease (Table 3). Based on the multivariate analysis, nab‐paclitaxel‐based regimens showed a significantly better pCR rate than paclitaxel‐based schemes (OR, 2.283; 95% CI: 1.161–4.490; *p* = 0.017). Also, the blood vessel invasion was a negative influencing factor of pCR (OR, 0.056; 95% CI, 0.007–0.428; *p* = 0.005; Table 3). Thus, the ICIs‐nabTP group could improve the postoperative pathological results, which may bring a new choice for neoadjuvant therapy of ESCC patients.

**TABLE 3:** Univariate and multivariate analysis of pCR in resectable thoracic ESCC

Variables	Univariate analysis			Multivariate analysis	
	Yes (%) (*n* = 57)	No (%) (*n* = 139)	*p*‐value	HR (95%CI)	*p*‐value
Age (<62 years)	23 (40.4)	68 (48.9)	0.275		
BMI (<23.3)	30 (52.6)	71 (51.1)	0.843		
T Stage			0.677		
cT1‐2	6 (10.5)	12 (8.6)			
cT3‐4	51 (89.5)	127 (91.4)			
N Stage			0.549		
cN0‐1	51 (89.5)	120 (86.3)			
cN2‐3	6 (10.5)	19 (13.7)			
Location			0.209		
Upper	11 (19.3)	24 (17.3)			
Middle	17 (29.8)	60 (43.2)			
Lower	29 (50.9)	55 (39.6)			
Grade			0.216		
G1	4 (7.0)	9 (6.5)			
G2	19 (33.3)	52 (37.4)			
G3	13 (22.8)	67 (48.2)			
Unknown	21 (36.8)	11 (7.9)			
Chemo interval (≤4 weeks)	13 (22.8)	27 (19.4)	0.594		
Chemo cycles (2–4)	53 (93.0)	133 (95.7)	0.672		
ICIs			0.102		
Camrelizumab	14 (24.6)	49 (35.3)			
Other (toripalimab, pembrolizumab or sintilimab)	43 (75.4)	90 (64.7)			
Platinum			0.336		
Cisplatin/nedaplatin	50 (87.7)	128 (92.1)			
Carboplatin	7 (12.3)	11 (7.9)			
PPI (yes)	17 (29.8)	38 (27.3)	0.725		
Chemo Drugs			0.018*	2.283 (1.161–4.490)	0.017*
Nab‐paclitaxel	36 (63.2)	62 (44.6)			
Paclitaxel	21 (36.8)	77 (55.4)			
Nerve invasion	1 (1.8)	26 (18.7)	0.002*	0.150 (0.018–1.232)	0.077
Blood vessel invasion	1 (1.8)	43 (30.9)	<0.001*	0.056 (0.007–0.428)	0.005*
LND (≤36)	39 (68.4)	112 (80.6)	0.076		
Pulmonary comorbid	8 (14.0)	16 (11.5)	0.624		
COPD	1 (1.8)	1 (0.7)	0.513		
Emphysema	2 (3.5)	9 (6.5)	0.413		
Other	5 (8.8)	6 (4.3)	0.374		
Cardiovascular comorbid	21 (36.8)	45 (32.4)	0.548		
Hypertension	21 (36.8)	38 (27.3)	0.188		
Coronary heart disease	4 (7.0)	7 (5.0)	0.584		
Arrhythmia	2 (3.5)	5 (3.6)	0.976		
Cerebral stroke	1 (1.8)	9 (6.5)	0.173		
Hepatic and renal comorbid	1 (1.8)	4 (2.9)	0.651		
Diabetes	4 (7.0)	8 (5.8)	0.738		

* *p* < 0.05.

## DISCUSSION

In the past decades, nCRT and nCT before surgery have constituted the mainstay of treatment for locally advanced EC.^28^, ^29^, ^30^, ^31^ Boonstra et al. previously demonstrated that overall survival (OS) was better in the nCT than in the surgery group (*p* = 0.03, HR0.71; 95% CI: 0.51–0.98).^29^ However, postoperative pulmonary complications were significantly more observed in the nCT group (*p* = 0.041).^29^ Based on the CROSS and NEOCRTEC5010 studies, compared with surgery alone, preoperative chemoradiotherapy could also obtain better OS. However, the French FFCD 9901 study does not demonstrate the survival benefits of nCRT combined with surgery, and in fact, the higher postoperative mortality cannot be neglected (11.1% vs. 3.4%, *p* = 0.049).^5^ In group nCRT, postoperative deaths are mainly caused by uncontrollable chylothorax, anastomotic leakage, gastric conduit necrosis, etc.^5^ Therefore, we aimed to explore multimodal neoadjuvant therapies for the treatment of locally advanced EC, which will not increase surgical complications and perioperative mortality.

Immunotherapy has developed rapidly in recent years, and also brings a glimmer of hope for advanced EC patients.^32^, ^33^, ^34^, ^35^ However, little is known about neoadjuvant immunotherapy for EC. Several clinical trials have been completed to explore the therapeutic effect of anti‐PD‐1/PD‐L1 plus nCRT for locally advanced EC: PALACE‐1 trial for ESCC, PERFECT trial for esophageal adenocarcinoma.^12^, ^13^ The PALACE‐1 trial showed that the combination of ICIs and nCRT yielded encouraging results with an exciting pCR rate of 55.6%,^13^ which is higher than the CROSS scheme (49%).^4^ However, in 13 of the 20 patients (65%), grade III and higher adverse effects (AEs) were observed.^14^ There was also no statistically significant difference in long‐term survival between the PERFECT and the nCRT cohort.^12^

At present, some clinical experiments conducted in the background of the Chinese population, such as the NICE‐2, NIC‐ESCC2019, TD‐NICE trials, confirm that immunochemotherapy has promising antitumor activity for resectable ESCC.^14^, ^15^, ^16^, ^17^, ^18^ TD‐NICE study participants responded positively to tislelizumab plus nabTP after three cycles, presenting a manageable safety profile and an encouraging pathological response (40.0%).^15^ Comparatively, the NCT04177797 study is a single‐arm, open‐label, phase II trial of locally advanced ESCC, in which patients received preoperative toripalimab plus TP schemes for two cycles. Only three (3/16, 18.8%) patients achieved pCR,^17^ indicating a potential that as a neoadjuvant treatment for ESCC, the effect of nabTP plus ICIs may be better than TP plus ICIs. Also, previous Chinese studies have confirmed that the nabTP versus TP had a higher objective response rate for advanced ESCC patients. Significant PFS improvement and less side effects are observed in favor of the nabTP arm.^26^, ^36^ A single‐arm trial evaluating nCT of nabTP for resectable ESCC also yielded positive results.^27^, ^37^ Here, we retrospectively compared the efficacy of ICIs with TP or nabTP for locally advanced ESCC.

Long‐term outcomes in multiple tumor types have been shown to benefit from pCR following neoadjuvant therapy.^38^, ^39^ Accordingly, pCR was defined as the main efficacy endpoint in our study. Our analysis of 196 surgical patients revealed encouraging results: the pCR rate was 36.7% (36/98) in the ICIs‐nabTP group and 21.4% (21/98) in the ICIs‐TP group (*p* = 0.018). We examined the influence of clinical characteristics on pCR using a multivariate analysis (Table 3). The results showed that vascular invasion or chemotherapeutic drugs were consistent with pCR (all *p* < 0.05). The pCR was more likely to occur in patients with nabTP plus ICIs (OR:2.283; *p* = 0.017). In addition, the 30‐day perioperative outcomes were comparable between ICIs‐nabTP and ICIs‐TP groups, in terms of the incidence of major pulmonary, cardiac, and anastomotic complications (Table 2, Figure 2). However, in the ICIs‐nabTP group, the incidence of 30‐day readmissions and hydropneumothorax were 4.1 and 11.2%, respectively and these rates were higher compared to ICIs‐TP group (Table 2). Thus, our center carried out a multicenter, randomized controlled study (ChiCTR2000040034). Patients in cohort A will receive two courses of cisplatin at 75 mg/m^2^, camrelizumab at 200 mg/bodyweight on day 1 and nab‐paclitaxel at 125 mg/m^2^ on days 1 and 8 every 3 weeks. Patients registered to a cohort B will be administered paclitaxel at 175 mg/m^2^ on the first day, then cisplatin and camrelizumab were the same regimens as cohort A. However, the comparative data needs further follow‐up.

A glucocorticoid, such as dexamethasone is widely used before chemotherapy of TP to eliminate side effects. However, its immunosuppressive impact is double‐edged. Some studies have found that preoperative dexamethasone administration can lead to a higher rate of recurrence in colon cancer^40^ and promote lung metastasis in breast cancer.^41^ This was a retrospective study. The detailed dosage and cycles of chemotherapy drugs, and the types of premedication before paclitaxel use, hormones and proton pump inhibitors, may be not exactly the same, which may have caused some bias to the results. Second, due to the short follow‐up time, the long‐term survival comparisons are lacking. Finally, some laboratory indicators related to adverse reactions were not recorded in detail by the surgical department. Therefore, a randomized controlled study is needed to further verify the results of our study.

In conclusion, the addition of nabTP to ICIs for locally advanced ESCC was feasible and safe without compromising surgical outcomes. As shown, the pCR rate was higher in the ICIs plus nabTP group than that in the ICIs‐TP group (*p* = 0.018), which may bring survival benefits for patients with ESCC.

## AUTHOR CONTRIBUTIONS

The design of the work: Yin Li. The acquisition of data: Yafan Yang, Haomiao Li, Yong Li, Zhen Wang, Qingfeng Zheng, Xiankai Chen. The analysis, or interpretation of data for the work: Yafan Yang, Haomiao Li, Peng Luo. Drafting the work or revising it critically for important intellectual content and final approval of the version to be published: Yafan Yang, Jianjun Qin, Yaxing Shen, Ruixiang Zhang, Xiaozheng Kang, Yin Li, Jie He. And the author “Yaxing Shen” pay the page fee for this article. Agreement to be accountable for all aspects of the work in ensuring that questions related to the accuracy or integrity of any part of the work are appropriately investigated and resolved: Yafan Yang, Yin Li.

## FUNDING INFORMATION

There was financial grants and funding support by the “Non‐profit Central Research Institute Fund of Chinese Academy of Medical Sciences (2019PT320022) and the China Postdoctoral Science Foundation (2018M631394)” for this article.

## CONFLICT OF INTEREST

The authors have no conflicts of interest to declare.
